# Younger and older chronic somatoform pain patients in psycho-diagnostics, physician-patient relationship and treatment outcome

**DOI:** 10.1186/1751-0759-7-4

**Published:** 2013-02-04

**Authors:** Bernd Bergander, Laurence Erdur, Bettina Kallenbach-Dermutz, Hans-Christian Deter

**Affiliations:** 1 Department of Psychosomatics and Psychotherapy, Charité Campus Benjamin Franklin, Hindenburgdamm 30, Berlin, 12200, Germany

**Keywords:** Chronic somatoform pain, Age, Psychosomatic in-patient treatment, Attachment style

## Abstract

**Introduction:**

Patients with chronic pain are found with highly variable clinical presentation and differing physical complaints. They are seen as a heterogenic group. Based on clinical observations, elderly patients seem to differ from younger patients with chronic pain. We examined whether there were systematic differences between young and old pain patients.

**Methods:**

As part of a routine evaluation of university hospital care, a newly developed psychosomatic treatment model for chronic somatoform pain disorders was examined. The basis for treatment efficacy was a target-oriented, specific somatic and psychological intervention that included a stable physician-patient relationship. Particular attention was paid to differences in treatment outcome with regard to changes in both physical and psychopathological symptom levels. We hypothesised that younger pain patients had higher psychological burden and benefitted more from our treatment than older pain patients.

**Results:**

Overall, 179 inpatients (57.5% women) with chronic pain were examined (age between 16 and 79 years). The group as a whole yielded high scores on the somatisation dimension (SCL-90) and showed a considerable amount of psychopathological symptoms, such as depressive mood and anxiety (HADS) and a great emotional instability (FPI-R). Age differences were only found with regards to patients’ degree of aggression (SCl-90): younger patients showed higher aggressive tendencies than older ones (p< 0.05). The treatment offered helped patients in both age groups especially with regard to reduction of depressive mood (HADS, p< 0.01) and anxiety levels (HADS, p< 0.01). Regression analysis showed different age groups and gender as significant predictors of anxiety reduction under therapy (R^2^=.108; model: p< 0.01).

**Discussion and conclusion:**

Results show that younger chronic pain patients suffer more from a considerable amount of psychological distress than older ones, but our treatment approach was equally effective in both groups. However, age and gender differences, as well as the patient’s baseline level of anxiety influenced the outcome. These factors need to be studied in future research.

## Introduction

Pain may present itself in different ways. It may be acute or chronic, somatic or psychogenic. How pain is perceived, experienced and managed depends not only on the organic pathology but also on the patients’ previous experiences, personal history, and personality structures. Chronic pain is a poorly understood condition and a challenge for both accurate diagnosis and adequate treatment. If pain becomes the central topic between physician and patient, it is often an indication for the beginning of a long journey of suffering.

### The diagnostic process

The primary concern for both physicians and patients is to localise the pain and its causes as quickly and reliably as possible. Assigning the manifest symptoms of the pain to a clear, underlying clinical cause is essential. Physicians’ diagnoses depend on the patient’s personal account and self-report; a comprehensive description of the experienced pain is thereby crucial.

Thus, a thorough medical history, including both the patient’s subjective description of all symptoms and the physician’s comprehensive physical examination, are needed. Once the patient can describe his or her pain coherently in terms of its chronological development, localisation, intensity and quality, a specifically targeted physical examination can begin. Only if the pain’s quality, intensity and localisation can be linked to its morphological and pathological origins can an adequate treatment approach be offered. A successful treatment may lead to considerable mitigation or even complete alleviation of the experienced pain. If these conditions are provided, a supportive and stable future physician-patient relationship can be expected. Often however, the diagnosis, treatment and medical outcomes in pain patients are unsuccessful. Although there is a high variability among pain patients, based on clinical presentations, we want to specify whether systematic trends and differences could be found among different subgroups of somatoform pain patients.

There are two groups of pain patients [1]. A first group consisting of patients who have an excessive affective association with their pain and often use catastrophic pictures to describe it. The intensity of the pain is often reported between seven and ten on a visual analogue scale without the occurrence of pain free intervals over many weeks or even months. A second group consists of patients, who in opposition to the first group, describe their pain without emotions and might thus be described as affectively dissociated from their pain.

Both verbal accounts of an experienced pain have the potential to compromise an adequate diagnosis. Given the complex mechanisms that underlie pain perception and its verbalisation, inadequate patient descriptions may lead to misinterpretation, frustration, anger or resignation on the part of the physician. This in turn can distort the diagnosis and may lead to the wrong treatment suggestions. Independently of biological, psychological and social factors, acute pain requires an immediate analgesic treatment, in order to alleviate suffering and prevent the pain from becoming chronic. However, despite successful treatment, pain is always affect-laden and relates to one’s previous experiences as well as to current circumstances. If a particular acute pain is accompanied by an existentially threatening event (such as redundancy/losing one's job, separation from partner, severe illness of a relative etc.) in conjunction with previously established risk factors (such as trauma or addiction), it may undergo a rather prolonged process, eventually becoming chronic with a high propensity to develop into a somatoform pain disorder. Patients suffering from somatoform pain disorder do not perceive physical and psychological symptoms, such as restlessness, fear, anger, sadness, but instead experience and communicate these as physical pain.

### Treatment of pain patients

We have made remarkable progress over the last few years with regard to the management and treatment of acute pain. Standardised analgesic treatment helps patients to bear acute pain after injury, peri-surgical, during labour, during myocardial infarction, tumour and during palliative care. Important thereby is physicians’ sufficient knowledge of available and appropriate analgesics, adequate application, dosage and frequency of administration. In addition, most hospitals (in Germany) have now an established pain clinic, where patients who do not respond to first-line analgesic pain relief can be referred. However, despite our increasing understanding of pain and its management as well as better provision of treatment available, many treatment attempts still remain unsuccessful. Pain associated with considerable difficult life events can become unmanageable and in that prevent a rigorous diagnosis explaining the underlying causes for the acute or chronic pain. Without the inclusion of bio-psycho-social factors into the treatment model and the understanding of physician patient relationship such pain management will not be successful.

The very first consultation shapes the physician-patient relationship and in that determines the nature and development of all future encounters. It offers the patient the opportunity to describe their experienced symptoms comprehensively. It is known that an accurate description of one’s experienced pain can be limited by or even be made completely impossible for a variety of reasons, including personal history, personality, peripheral and central pain processing ability, and other previous experience [1]. Thus, misconceptions in the form of false expectations, worry, resentment and disappointment are bound to emerge.

They can have serious negative consequences for the quality and nature of the patient-physician interaction. Patients experiencing pain often expect to be treated quickly and effectively, with a wish that it leads to long lasting pain relief. In addition to other implicit or unconscious expectations, expectations and demands are also made by patients’ relatives, their socio-economic environment, their social insurance agencies and employer; all of which have an impact on the physician-patient relationship. Finally, the physician’s own ambitious aims should not be forgotten, as they play a pivotal role in the development and management of the interaction between both parties. Thus, it becomes clear that a complex mixture of conditions shape and equally strain the relationship between the physician and the patient. The first encounter between both and how the clinical assessment is carried out plays a pivotal role in the subsequent treatment approach. It determines all future expectations and within that sets the frame for a successful treatment. Whether or not the treatment of pain is successful depends on how supportive the interaction is being perceived by the patient. Thus a physician’s ability to adjust to the patient’s individual history and personality is of tremendous importance. A positive treatment alliance is a positive precursor, yet the very nature of pain often brings about particular challenges so that to date the treatment of pain patients remains a clinical challenge [2]. Pain, which is experienced in conjunction with feelings of insecurity, threat or fear, activates a particular coping mechanism, namely our attachment system with the need for safety, security and protection. This system, once activated, also assumes a key role in the adaptation and management of acute and chronic pain [1]. Thus, a secure physician-patient relationship is necessary for lasting symptom reduction and pain relief. The interaction between physician and patient often is complicated by misunderstandings that are difficult to rectify and thus may lead to an inadequate therapeutic alliance. Besides offering a stable and reassuring therapeutic environment, expectations and realistically achievable goals on the part of both physician and patient need to be formulated.

### Treatment outcome - three groups of patients can be found

1. “Responder” are those, who respond to a therapy as usual (TAU) -pain management successfully.

2. “Partial responder” are those who benefit from pain management but do not experience complete pain relief. They are, however, able to continue or adapt their lifestyle accordingly.

3. “Non-responder” do not respond to TAU - treatment at all. The pain levels in the latter group remain unchanged and are often experienced as catastrophic. Medication does not work as expected and often leads to disagreeable psychotropic side effects, such as extreme sedation and euphoria, and thus carries high potential for a subsequent addiction. A somatic-morphological assessment cannot be made in the last group [3].

Especially, the last group shows important consequences in the physician-patient relationship. The aforementioned expectations of a treatment resulting in immediate pain release has not happened, thus leading to feeling misunderstood and further feelings of disappointment, distrust, fear, helplessness, rage and hurt. These experienced secondary emotions change the patient-physician relationship. Thus, two diametrically opposed treatment processes may develop. Either an unreasonably invasive assessment procedure and treatment is carried out despite unsuccessful results, or physician and patient do not manage to achieve an adequate assessment procedure and subsequent treatment at all. Both, however, trigger a vicious cycle leading to the pain becoming chronic. These kinds of patients are often transferred to interdisciplinary pain conferences of university medical centres or psychosomatic in-patient treatments in German cities.

The difficulties in treating chronic pain patients successfully led to the important clinical question of if particular sub-populations of pain patients - transferred to psychosomatic inpatient treatment - react differently as some have argued [2]. Do age, gender and degree of the condition differ amongst the different pain outcome groups given? Children compared to teenagers show a significant difference in terms of pain perception and subsequent treatment [4]. Our impression so far is that younger patients possess better preconditions for effective treatment as shown in greater pain reduction and reduced psychological symptoms, such as anxiety and depression. It is much more difficult to gain access to psychological aspects of the experienced pain if the patient’s sole focus is on morphological dysfunction. This process of integrating psychological origins of pain in the thinking of patients seems also dependent on the patient’s age. Further reasons for an impeded access to possible underlying psychological causes is an inability to perceive as well as tolerate emotions, an incapacity for introspection, or past experiences of rejection, which may distort the therapeutic relationship in the here-and-now. Older individuals seem to have greater deficits and fewer possibilities to verbalize their experiences in this area [5]. To our knowledge this clinical finding has only been analysed and shown in older patients [5], but no comparison between younger and older patients has been done within a psychosomatic inpatient treatment model.

We posed the following hypotheses within the clinical evaluation study: Firstly, we stated that younger pain patients have a significantly lower pain threshold (a) and more psychological (b) and management problems (c) than older pain patients. It follows that a psychosomatic treatment would be more effective for the former group. Secondly, we stated that our treatment approach had an effect on our pain patients as demonstrated in symptomatic change on the depression (a) and anxiety scale (b) of the HADS.

## Methods

Preliminary remark: All patients who experienced physical and/or psychological pain as their chief complaint for more than six months were given the acronym ‘pain patient’ in this article. Initially, we included all patients who expressed suffering from chronic pain and were referred to our unit for its treatment. All of them had an initial assessment that included a comprehensive physical examination and history-taking in line with a bio-psycho-social approach. Further diagnostic differentiation was carried out through examinations in different medical specialties of the university medical centre interdisciplinary in a second step.

### Design

We conducted a retrospective analysis based on our clinical documentary system which we apply for all inpatients treated in our department. Questionnaires are handed to the inpatient at the beginning and at the end of treatment, consisting of a questionnaire battery suitable for psychosomatic patients. Results are calculated by a psychologist and also examined by the treating psychotherapist. Moreover, medical diagnoses of the inpatients are documented at beginning of treatment and after discharge of the patient.

### Subjects

179 patients with chronic pain were treated at the psychosomatic unit of the university hospital Charité Campus Benjamin Franklin, Berlin between 2000 and 2008. Of these inpatients 115 were women and 61 were men with a mean age of 50 years (range 16–79 years). The average inpatient stay was 25 days (sd 10 days). Most patients had been seen at the hospitals’ interdisciplinary pain clinic, which receives referrals from general practitioners and specialist consultants (orthopaedics, neurologists, and psychiatrist) within the South-Berlin catchment area.

All patients with chronic somatoform pain were included in the piloting of our treatment, independently of whether other diagnoses, such as chronic back pain, low back pain, functional gastro intestinal pain, muscle-skeletal pain, headaches or severe physical impairment were given. The diagnosis in accordance with the ICD-10 diagnostic criteria was carried out by an interdisciplinary workgroup, which consisted of psychosomatic clinicians, specialised pain therapists, internists, and neurologists.

To understand differences of pain patients according age a median split was performed on the total number of patients in order to differentiate between two comparable age groups. This yielded a “young patient group” ranging from 16–49 years of age and an “old patient group” ranging from 50–79 years of age. In a second step we examined in a multivariate analysis the whole range of age and other factors like gender and psycho diagnostic variables. Given the small sample size of patients and the lack of questionnaire data to examine attachment styles, we focused in this first pilot study only on the two age groups.

### Psychiatric diagnosis

Psychiatric diagnoses were made following the ICD-10 diagnostic criteria at the first week of the inpatient treatment. All 179 patients were diagnosed with the diagnosis persistent Somatoform pain disorder (SPD; F45.4); 128 (71.5%) of the 179 patients SPD as the main diagnosis. The remaining 51 (28.5%) received a diagnosis of somatic disorder, with 32 (17.9%) being given SPD as primary, 17 (9.5%) as secondary and 2 (1.1%) as tertiary diagnosis. Additional psychiatric diagnoses we found in ICD-10 chapter F0 (n=1), F1 (n=39), F1 (n=2), F3 (n=50), F4 (n=47), F5 (n=24), and F6 (n=10). Within the inpatient treatment we were able to diagnose a small group of patients with a severe somatic disease whilst they were initially referred to us with a diagnosis of psychological pain.

### The pilot treatment

Out therapy model is described as an integrative, psychosomatic treatment. It is described in great detail by Wulsin et al. (2005) [6]. Therefore, we will only summarise it here briefly. It consists of “simultan-diagnostic”: Focus was to differentiate the pain diagnosis, e.g. exploration of pain, circumstances/events at the time of the exacerbation of the pain, acknowledgment of the burden caused, and exploration of the psychopathological assessment. Focus and aims of the inpatient pain treatment (“simultan-therapy”) was to summarise assessment results, develop a treatment plan, to discuss a time-line, and to consider a proposal of the relationship and individual personality structure of the patient. The standard pain management program was complemented with individual psychotherapy (three-times weekly 30 min), group psychotherapy (once-weekly 90 min), guided relaxation exercises (three-times weekly), once weekly art and concentrative movement therapy as well as once weekly pain-management groups.

### Assessments

Anxiety and Depression was assessed using the German version of the Hospital Anxiety and Depression Scale (HADS; 9). The self-report questionnaire was administered pre and post treatment. The instrument consists of two subscales, one for anxiety and one for depression with maximum scores of 21 on each subscale. The HADS is a well established instrument to measure anxiety and depression in in-patients [7].

Subjective impairment due to physical and psychological symptoms and the alteration of impairment were determined using the Symptom Checklist (SCL-90-R; by [8]; Hardt et al. used a version for pain patients;) [9]. Patients completed the self-report questionnaire before treatment began. The self-report measure consists of nine scales: depression, anxiety, somatisation, obsessiveness, insecurity in social contacts, aggressiveness/hostility, phobic anxiety, paranoid tendencies and psychoticism. Psychological distress was assessed using the Global Screening Scale (GSI; 8). Distress values were defined by scores above a cut-point of T ≥ 60.

Personality dimensions were assessed using the Freiburg-personality-inventory (FPI-R; 10). The self-rated instrument consisting of 137 questions that patients are asked to score as either “true” or “untrue”. The answers are compiled into 12 scales or personality dimensions. These include: satisfaction with oneself, social orientation, need for achievement, inhibition, irritability, aggression, demandedness, physical complaints, worries about health, openness, extraversion and emotionality. With regards to the described personality traits of pain patients [10], the scales physical complaints, emotionality and aggressiveness were of particular interest to us. We used the FPI-data analysis program (SPSS). Stanine values were standardised according gender and age.

In addition, a standardised questionnaire for collecting patient medical history was used. All measures were administered during the first three days of admission and once again, (with the exception of the SCL-90 ) during the last three days of their in-patient stay before discharge.

### Statistical analysis

Descriptive statistics are used for a detailed description of the patients. All data are presented as mean (M) and standard deviation (SD) or the absolute and relative frequencies for categorical data.

Differences between the age groups were analyzed using the Mann-Whitney’s U test or Wilcoxon’s signed rank test for comparisons between different time points. The Chi square test was used for group comparisons involving categorical data. Fisher’s exact test was used for cases where more than 25% of the cells had an expected cell size of less than five.

To test whether the age had an effect on depression or anxiety reduction a one way ANOVA was used. We controlled for gender and age in subgroups. Coefficients were considered significant if the respective p-values were less than α = 0.05.

To examine predictors for depression and anxiety reduction, we conducted a logistic regression and included age, gender and severity of disease as independent variables in a multivariate model using stepwise inclusion of parameters.

Calculations were done with SPSS 19.0. Coefficients were considered significant if the respective p-values were less than α = 0.05.

## Results

Table 1 provides a summary of anamnestic and demographic data for both age groups. Overall, the results show no significant differences between the two groups with regards to gender, somatic main diagnosis, and length of in-patient stay. Differences were found with respect to some socio-economic status items and analgesic medication use. Younger patients were more often single (p< 0.01) or left school without graduation more often (p< 0.02) but had higher employment rates (p< 0.001) compared to older patients. In agreement with our hypotheses 1c, duration of stay was higher in the younger age group:, but this was not significant (Table 1). Analgesic medication consumption was found to be higher in older patients than in younger ones (n.s.).

**Table 1:** Anamnestic and social data of younger (N= 90) and older (N= 87) chronic somatoform pain patients (Chi square test)

	**Total group (N=177)**		**16-49 years old (N=90)**		**50–79 years old (N=87)**		**Chi square**
	**N**	**%**	**N**	**%**	**N**	**%**	**p**
Gender (% female)	115	65	57	65.5	57	65.5	n.s
**School education**							*
no graduation	14	7.8	11	12.3	3	3.4	
special education	3	1.7	3	3.3	0	0	
Elementary school/secondary school	39	21.8	14	15.6	25	28.7	
secondary school/polytech education	62	34.6	29	32.2	33	37.9	
A-levels	29	16.2	17	18.9	12	13.8	
**Marital status**							
single	24	13.4	21	23.3	3	3.4	***
married, living with partner	93	52.0	38	42.2	55	63.2	
divorced/separated	24	13.4	11	12.2	13	14.9	
widowed	4	2.2	2	2.2	2	2.3	
**Professional education**							n.s
In training	2	1.1	2	2,2	0	0	
apprenticeship/vocational school	73	40.8	31	34.4	42	48.3	
polytech/college of higher education	30	16.7	15	16.6	15	17.2	
without professional education	24	13.4	17	18.9	7	8.0	
**Current employment status**							****
full-time employment	27	15.1	19	21.1	8	9.2	
part-time employment	12	6.8	7	7.8	5	5.6	
housewife/househusband	10	5.6	5	5.6	5	5.7	
student/in education	6	3.4	6	6.7	0	0	
registered unemployed	35	19.6	22	24.4	13	14.9	
disability pension	11	6.2	4	4.4	7	8.0	
early retirement	31	17.3	3	3.3	28	32.1	
other	8	4.5	3	3.3	5	5.7	
**Analgesic consumption**							n.s
No	37	20.9	22	36.7	15	28.3	
< 1/week	8	4.0	5	8.3	2	3.8	
1-3/week	13	7.3	9	15.0	4	7.5	
> 3/week	56	32.2	24	40.0	32	60.4	
**Duration of inpatient treatment**							n.s
< 1 week	9	5.1	4	4.5	4	4.6	
1-2weeks	15	8.5	7	6.8	9	10.3	
2-3 weeks	33	18.6	17	18.2	16	18.4	
3-4 weeks	52	29.4	28	31.8	24	27.6	
> 4 weeks	61	34.5	30	34.1	31	35.6	

*= p< .05, **= p< .01, ***=p < .001, ****=p < .0001.

Overall, psychological assessment at admission revealed high pathological scores in “physical complaints”, “emotional instability” (> 6.0 stanine on the FPI), “somatisation”, (> 60 T value on the SCL 90) and “depression” and “anxiety” (> 9.8 points on the HADS), but they were similar to those of normal control samples in the other examined psychological dimensions (Table 2). In agreement with our hypotheses 1b, the age groups differed in psychological dimensions: younger patients showed higher “aggression” (SCL 90, p= 0.03) and a statistical tendency toward a higher “depression” score (HADS, SCL-90) and in the mean more phobic anxiety (SCL 90). But, against our hypothesis 1a, we found mean lower somatisation (SCL 90) in the younger group (n.s).

**Table 2:** Differences between younger (N= 90) and older (N= 87) chronic somatoform pain patients (t- test): the Freiburg Personality Inventory and the SCL-90 R before treatment

**Measures**	**Total group (N=177)**		**16-49 years old (N=90)**		**50-79 years old (N=87)**		**t-test**
***Freiburg Personality Inventory (FPI)***							
Pre-treatment (Stanine Score)	m	sd	m	sd	m	sd	p
Satisfaction with oneself [1]	3.4	1.7	3.3	1.7	3.5	1.7	n.s
Social orientation [2]	5.8	1.6	5.8	1.6	5.8	1.6	n.s
Need for achievement [3]	4.4	1.7	4.4	1.8	4.4	1.6	n.s
Inhibition [4]	5.9	4.9	6.4	6.7	5.4	1.7	n.s
Irritability [5]	5.7	2.0	5.4	2.0	5.9	2.0	n.s
Aggression [6]	4.7	1.9	4.6	1.8	4.6	1.9	n.s
Demandedness [7]	5.8	1.6	5.7	1.7	5.9	1.4	n.s
Physical complaints [8]	6.5	1.6	6.6	1.4	6.3	1.8	n.s
Worries about health [9]	4.5	1.7	4.7	1.8	4.4	1.6	n.s
Openess [10]	4.5	1.8	4.5	1.8	4.5	1.7	n.s
Extraversion [11]	4.1	1.7	4.0	1.7	4.3	1.6	n.s
Emotional instabilityity [12]	6.4	1.5	6.5	1.5	6.3	1.8	n.s
***Symptomchecklist SCL-90 R (T-Score)***							
Psychoticism	58.7	11.1	59.5	10.6	57.9	11.5	n.s
Paranoid thinking	53.3	12.8	54.5	13.4	52.1	12.1	n.s
Phobic anxiety	58.0	13.3	59.1	14.7	56.9	11.9	n.s
Aggression	55.7	12.1	57.9	12.5	53.4	11.3	*
Anxiety	61.2	12.8	62.1	12.9	60.3	12.8	n.s
Depression	63.4	12.7	64.9	12.6	61.8	12.7	n.s
Insecurity	54.7	12.5	56.6	12.8	52.8	12.1	n.s
Obsession	59.1	14.3	59.9	14.4	58.2	14.3	n.s
Somatization	67.4	13.0	66.3	12.9	68.4	13.0	n.s
GSI	63.0	13.5	64.0	13.9	62.0	13.2	n.s
PSDI	64.1	11.4	64.5	12.1	63.8	10.6	n.s
PST	59.7	13.7	60.7	13.7	58.6	13.7	n.s

*GSI*= Global Symptom Index (Total Score), *PSDI*= Index of response behaviour.

*PST*= number of positive responses.

* =p< .05.

Based on clinical reports of the ward physicians, two thirds of the participants profited from treatment in relation to experienced pain symptoms and perceived psycho-education regarding possible causes. For a summary of the patient report, the pre and post treatment HADS was analysed. As shown in Table 3, it was found that both anxiety and depression scores were significantly reduced after treatment for both age groups.

**Table 3:** Differences between younger (N= 90) and older (N= 87) chronic somatoform pain patients in the Hospital Anxiety and Depression Scale (HADS) before and after treatment (t-test) and in the course of time (Wilcoxon test)

	**Total group (N=177)**		**16-49 years old (N=90)**		**50-79 years old (N=87)**		**t-test**
HADS	m	sd	m	sd	m	sd	p
Anxiety_pre-therapy	9.7	4.5	9.8	4.5	9.6	4.6	n.s
Anxiety_end of therapy	8.4	4.9	8.3	4.5	8.1	5.3	n.s
Change of anxiety (end of therapy - pre-therapie)	1.4**	3.9	1.5**	0.6	1.5**	0.7	
Depression pre-therapy	10.3	6.0	10.7	4.6	10.0	7.2	n.s
Depression end of therapy	8.5	5.3	8.8	5.2	8.1	5.5	n.s
Change of depression (end of therapy - pre-therapie)	1.6***	3.4	1.9**	0.7	1.9**	0.7	n.s

Wilcoxon test: * p< .05, ** p< .01, *** p< .001.

Multivariate analysis where age, gender and diagnosis (somatoform vs somatic) were entered as covariates showed no significant changes of depression and anxiety scores. Regression analysis with the target variable changes of the depression/anxiety score during inpatient treatment showed no significant effects for age, gender and diagnosis. For anxiety, a significant effect of the baseline anxiety value was found (β =.30, p=0.001). This model, including anxiety baseline, age group (19–49 years vs. more than 49 years) and gender explained 10.8% variance (R^2^=.108) and was significant (p=0.005). The baseline effect was not significant for depression and the depression model showed no significance (R^2^=.017).

## Discussion

Findings of the present study highlight the heterogeneity of pain patients treated at our psychosomatic in-patient unit. As the descriptive analysis shows, one third (28.5%) were given a primary diagnosis of somatic disorder. Primary diagnosis was equally distributed across both younger and older patient groups. It could be argued that for no other disorders the bio-psycho-social diagnostic model and the acknowledgement of the complexity involved in the physician-patient relationship is of such importance, which might explain the diversity of diagnoses given to patients suffering with pain [2,3]. It is precisely in order to emphasise the phenomenon pain that we kept the undifferentiated term ”pain patients” despite the question raised as to whether or not it is appropriate to speak of such a group [11].

Chronic pain patients signify a considerable problem in the health care system [1,12].

Our results have shown that the majority of these patients were women between 16 and 79 years of age. These results confirm findings from other studies, in that patients showed a high degree of somatisation, reported a considerable amount of physical as well as psychopathological symptoms, and scored high on depressive mood, anxiety and emotional liability. Nickel et al., 2010 [2], for example, reported that 75% of their sample (282 psychosomatic pain inpatients) were female and that 69% of those reported a co-morbid psychological disorder. Furthermore, they found that the intensity of the somatisation as well as reported lower quality of life increased with age. Here, we were, however, unable to confirm these findings as the present results were found to be statistically non-significant, but went in the same direction (somatisation scale, SCL-90). An interesting finding from our study is that younger patients were found to be single, in employment, and reported having a better standard of knowledge despite a lower number of graduations than older patients. 7% of the younger patients compared to 40.1% of the older patients in our sample dropped out. The previously mentioned Mainz-Study reported that only16.1% of their participants dropped out.

When we looked at the other dimensions of the age sub-groups, only marginal differences were found with regards to pain experience, psychological symptoms, and disease management.

Differences between the two age groups were found with regards to reported levels of aggression, the mean duration of stay in hospital, and the mean consumption of analgesic medication. The significance of aggression in pain patients was also emphasised in studies about fibromyalgia patients [13].

A newly-developed integrative, psychosomatic treatment model was piloted for a group of chronic pain patients that are routinely referred to our university clinic for in-patient treatment. 179 patients received the new treatment and preliminary results as to its effectiveness are outlined by a descriptive analysis of the patient group. Two-third of the patients with a primary diagnosis of somatoform pain disorder benefited from our treatment, as shown in a reduction of symptoms of depression and anxiety.

Opposed to our expectations, both age groups benefited from treatment. No significant differences were found, which was surprising [14] given that previous findings report older patients to benefit less from psychotherapeutic treatments [5,15]. Neither diagnosis nor gender influenced treatment efficacy in terms of depressive symptom reduction. But, anxiety symptom reduction was influenced by age group as well as gender. The symptom severity of anxiety at the beginning of treatment was also found to have an effect. The 10.8% detection of variance is not so high, but the model was significant in demonstrating that these factors are important in the in-patient treatment of chronic somatoform pain patients. In line with previous findings [16], the higher symptom severity, the better they benefited from treatment.

An additional note concerning psychodynamic aspects of pain treatment: The role of attachment was only integrated in the kind of treatment of pain patients. In this study, we could not demonstrate data for this important aspect [17,18]: It is often found that the physician-patient relationship reflects the early mother-infant attachment of the patient. According to the Bowlby (1969) [19] theory, children are born with a deep-seated, instinctive need for attachment to a primary care-giver. Attachment according to his theory means safety, warmth, availability and the provision of nourishment on the one hand, and appropriate stimulation for an exploration of the external environment without fear of losing the crucial bond on the other. Thus, an internal attachment organisation develops gradually in relation to the primary attachment figure. However, under certain circumstances, the failure of a secure attachment organisation may lead to an internal belief that reliance on a person for attachment is futile. In one where pseudo-autonomy is assumed, the individual tries to maintain an insurmountable distance. These infantile internal survival strategies are often reflected in the patient-physician relationship [20]. Patients with an internal secure attachment organisation are generally able to describe their symptoms, and any affective component usually corresponds to its content (21). Both, as said above, provide good conditions for an accurate diagnosis and subsequent treatment approach. Patients with an insecure attachment organisation, on the other hand, have difficulties in relating to their physician. Descriptions and associated affects often do not correspond, thus leading to an inaccurate verbal as well as non-verbal account of the experienced pain (21). In our study, we did not find that younger pain patients were different compared to older ones according attachment styles. In both groups the physical pain of these individuals becomes the sole focus and means of communication. Given our knowledge of these highly complex processes, within the therapy we tried to facilitate a trusting and emotionally corrective physician-patient relationship, which offered care and security, and thus enabled the patient to become aware of the underlying conflicts and finally allow the emotional discharge of formally only verbally expressed affects.

Limitations of the study: The described therapy model is based on the long-standing experience of individual physicians working at our clinic, all of whom differ in their approach to diagnosing and treating pain patients. Within the scope of this preliminary evaluation, we were able to describe in detail only clinical patients. This does not allow generalization of the results to all pain patients. However, the diagnosis in accordance with the ICD-10 diagnostic criteria was carried out by an interdisciplinary workgroup. They consisted of psychosomatic clinicians, specialised pain therapists, internists, and neurologists. Given the large variability within the somatic disorders in general, we have only focused on ICD 10, F 45.4 as the main diagnosis. The heterogeneity with regards to somatic disorders as primary and psychiatric disorders as a secondary diagnosis in relation to gender and age effects [2] were thus considered only broadly. Given that we are reporting the results of our routine evaluation, we lack the inclusion of pain-specific psychological as well as other psychosomatic outcome measures. A further limitation is the lack of a comparison or control group. Finally it can be questioned whether the age groups in this study provide the whole picture of age effects.

### Outlook

Inspite of these limitations we were able to describe sociological, clinical and psychological differences between age groups in our examined pain patients. Additionally we could evaluate effects of the in-treatment in both groups, which were not different. This clinical evaluation study has shown there are differences between age groups, gender and somatics compared to somatoform pain diagnoses groups in chronic pain patients. This will need to be evaluated in the next few years to find out the best treatment strategies for “non responder patients”. In these cases we need a common view on the subjective experience and the individual disease management of patients. Pivotal is the exploration of the patient’s experience of pain. In order to gain a better understanding of the experience of pain patients, we have found it important not only to pay clinical attention to the individual but also to differentiate between different subgroups. This view, in conjunction with the basic understanding of pain management, is needed when dealing with with each patient as an individual suffering from pain.

## Competing interests

The authors declare that they have no competing interests.

## Authors’ contributions

BB drafted and wrote the manuscript, LE conducted statistical analysis, BKD and HCD conceptualized the study and revised the manuscript. All authors read and approved the final manuscript.
